# Poly[(2,2′-bipyridine-κ^2^ *N*,*N*′)(μ_3_-2,4,6-trimethyl­isophthalato-κ^5^ *O* ^1^,*O* ^1′^:*O* ^1^:*O* ^3^,*O* ^3′^)cadmium]

**DOI:** 10.1107/S1600536811054183

**Published:** 2011-12-23

**Authors:** Shao-Gang Hou, Mei-Fang Jin

**Affiliations:** a College of Chemical and Environmental Engineering, Anyang Institute of Technology, Anyang 455000, People’s Republic of China

## Abstract

In the crystal structure of the polymeric title complex, [Cd(C_11_H_10_O_4_)(C_10_H_8_N_2_)]_*n*_, the Cd^II^ cation is chelated by one 2,2-bipyridine ligand and two carboxyl groups from two trimethyl­isophthalate (TMIPA) anions, and is further coordinated by one carboxyl­ate O atom from a third TMIPA anion, forming a distorted penta­gonal–bipyramidal geometry. Each TMIPA anion bridges three Cd^II^ cations, forming polymeric complex sheets parallel to (001). Weak C—H⋯O hydrogen bonding occurs between adjacent sheets.

## Related literature

For applications of functional metal-organic frameworks, see: Evans & Lin (2002 ▶); Chen *et al.* (2010 ▶); Leong & Vittal (2011 ▶); Sun *et al.* (2011 ▶). For related structures, see: Ma *et al.* (2008 ▶); Zhang *et al.* (2008 ▶); Zhou *et al.* (2003 ▶); Zhang *et al.* (2003 ▶); He *et al.* (2010 ▶); Liu *et al.* (2008 ▶). For our previous work, see: Dai *et al.* (2008 ▶, 2009 ▶); Zhao *et al.* (2009 ▶). 
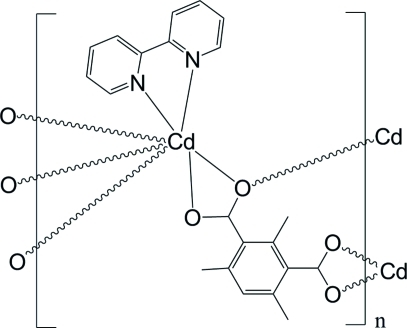


## Experimental

### 

#### Crystal data

[Cd(C_11_H_10_O_4_)(C_10_H_8_N_2_)]
*M* *_r_* = 474.77
Orthorhombic, 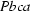

*a* = 13.1985 (8) Å
*b* = 15.5714 (9) Å
*c* = 18.1926 (11) Å
*V* = 3738.9 (4) Å^3^
*Z* = 8
Mo *K*α radiation
μ = 1.20 mm^−1^
*T* = 298 K
0.15 × 0.10 × 0.10 mm

#### Data collection

Bruker SMART APEXII CCD diffractometer
Absorption correction: multi-scan (*SADABS*; Bruker, 2001 ▶) *T*_min_ = 0.841, *T*_max_ = 0.890
14349 measured reflections
4299 independent reflections
2454 reflections with *I* > 2σ(*I*)
*R*_int_ = 0.054

#### Refinement

*R*[*F* ^2^ > 2σ(*F* ^2^)] = 0.045
*wR*(*F* ^2^) = 0.098
*S* = 0.99
4299 reflections
253 parameters
H-atom parameters constrained
Δρ_max_ = 0.51 e Å^−3^
Δρ_min_ = −0.63 e Å^−3^

### 

Data collection: *APEX2* (Bruker, 2007 ▶); cell refinement: *SAINT* (Bruker, 2007 ▶); data reduction: *SAINT*; program(s) used to solve structure: *SHELXS97* (Sheldrick, 2008 ▶); program(s) used to refine structure: *SHELXL97* (Sheldrick, 2008 ▶); molecular graphics: *DIAMOND* (Brandenburg, 2008 ▶); software used to prepare material for publication: *publCIF* (Westrip, 2010 ▶).

## Supplementary Material

Crystal structure: contains datablock(s) I, global. DOI: 10.1107/S1600536811054183/xu5394sup1.cif

Structure factors: contains datablock(s) I. DOI: 10.1107/S1600536811054183/xu5394Isup2.hkl

Supplementary material file. DOI: 10.1107/S1600536811054183/xu5394Isup3.mol

Additional supplementary materials:  crystallographic information; 3D view; checkCIF report

## Figures and Tables

**Table 1:** Selected bond lengths (Å)

Cd1—N1	2.393 (4)
Cd1—N2	2.334 (4)
Cd1—O1	2.607 (3)
Cd1—O2^i^	2.317 (3)
Cd1—O2	2.372 (3)
Cd1—O3^ii^	2.347 (3)
Cd1—O4^ii^	2.396 (3)

Symmetry codes: (i) 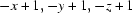; (ii) 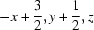.

**Table 2:** Hydrogen-bond geometry (Å, °)

*D*—H⋯*A*	*D*—H	H⋯*A*	*D*⋯*A*	*D*—H⋯*A*
C4—H4*A*⋯O1^iii^	0.93	2.32	3.240 (6)	169
C8—H8*A*⋯O3^iii^	0.93	2.39	3.251 (6)	155

Symmetry code: (iii) 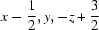.

**Fig. 1.**
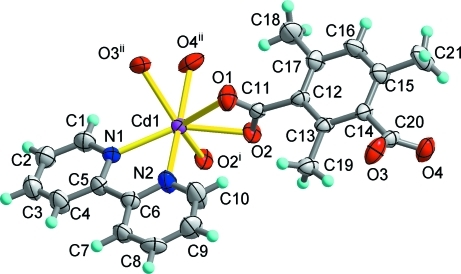
The structure of (I), showing the atom-numbering scheme and the coordination environment around the CdII centre. Displacement ellipsoids are drawn at 50% probability level and H atoms are shown as small spheres of arbitrary radii. (symmetry codes: (i) -x+1, -y+1, -z+1; (ii) -x+3/2, y+1/2, z)

**Fig. 2.**
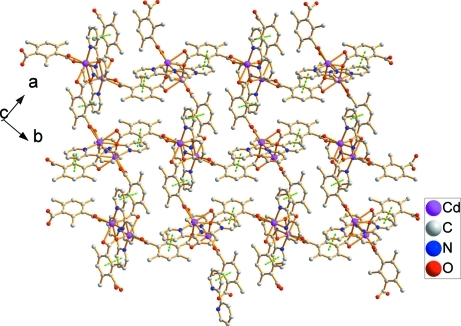
A ball-and-stick perspective view of the 2D sheet incorporating weak face-to-face π···π interaction (green dashed lines). Hydrogen atoms are omitted for clarity.

## supplementary crystallographic information

### Comment

The rational design and synthesis of functional metal-organic frameworks (MOFs) is a more and more fascinating field in recent years due to their interesting topologies and potential applications in gas adsorption, nonlinear optics, magnetism, molecular recognition, etc (Evans & Lin, 2002; Chen, *et al.*, 2010; Leong & Vittal, 2011; Sun *et al.*, 2011). As we know, the construction of MOFs mainly depends on the coordination geometry of metal ions and the nature of ligands. Besides, some secondary interactions, such as aromatic π···π interactions, classical hydrogen bonds (such as O-H···O and N-H···O hydrogen bonds), and non-classical hydrogen bonds (such as C-H···O hydrogen bond) often influence the packing of molecules from discrete subunits or low-dimentional entities to high-dimentional supramolecular frameworks. One of the most effective strategies to assemble MOFs is to use carboxylates as linkers because of their diverse conformations and coordination modes observed in the coordination process (Ma *et al.*, 2008; Zhang *et al.*, 2008). In spite of isophthalate-based MOFs (Zhou *et al.*, 2003; Zhang *et al.*, 2003) have been widely reported, to the best of our knowledge, only one MOF based on 2,4,6,-trimethylisophthalic acid (H_2_TMIPA) has been documented until now (He *et al.*, 2010). Based on our previous work (Dai *et al.*, 2008; Dai *et al.*, 2009; Zhao *et al.*, 2009) and consideration the steric hindrance effects of additional three methyl groups on isophthalate, herein, we choose the H_2_TMIPA as a bridging ligand to construct a novel Cd^II^ coordination polymer (I), which is a 2D (4,4) net incorporating [Cd_2_(COO)_4_N_2_] SBUs.

The asymmetric unit of (I) contains one crystallographically independent Cd^II^ center, one TMIPA ligand and one bpy ligand. The Cd^II^ ion is in a slightly distorted pentagonal bipyramidal geometry, completed by five O atoms from three different TMIPA ligands and two N atoms from the same bpy ligand (Fig. 1). The equatorial plane of pentagonal bipyramid is defined by O2^i^, O3^ii^, O4^ii^, N2 atoms, while the axial positions are occupied N1, O1 atoms. [symmetry codes: (i) -x+1, -y+1, -z+1; (ii) -x+3/2, y+1/2, z]. Two carboxyl groups on TMIPA ligand adopt two different coordination patterns, µ_1_-η^1^: η^1^ chelating and µ_2_-η^2^: η^1^ bridging, respectively. The Cd-N bond lengths are 2.393 (4) and 2.334 (4) Å, while the Cd-O bond lengths vary from 2.317 (3) to 2.607 (3) Å (Table). The average Cd-N and Cd-O distances in (I) are comparable with those reported for Cd-based MOFs (Liu *et al.*, 2008). Two crystallographically equivalent Cd^II^ anions are bridged by two tridentate bridging carboxyl groups to form a binuclear SBU with a Cd···Cd contact of 3.7886 (6) Å. Because of the steric hindrance between the methyl and the carboxyl groups, the two carboxyl groups of H_2_TMIPA are not coplanar with the central benzene ring, generating two dihedral angles of 55.1 (2) and 85.3 (2)^o^, respectively. The [Cd_2_(COO)_4_N_2_] SBUs are joined by TMIPA ligands to form an infinite 1D zigzag chain. Furthermore, TMIPA ligands connect the zigzag chain to a 2D layer (Fig. 2) which is consolidated by the intrasheet weak face-to-face π···π interaction between bpy and TMIPA with *C*g1···*C*g2^i^ separation of 3.725 (3) Å (Cg1 and Cg2 are the centroids of the N1/C1–C5 and C12–C17 rings, respectively; symmetry code: (i) -x+1, -y+1, -z+1). The bpy ligand acts as a terminal group to occupy the remaining coordinate sites, which prevents the structure from higher dimensionalities. The adjacent 2D layers are further extended to a 3D supramolecular framework by virtue of non-classical C-H···O hydrogen bonds (Table 2).

### Experimental

A mixture of Cd(NO_3_)_2_.6H_2_O (30 mg, 0.12 mmol), 2,4,6,-trimethylisophthalic acid (H_2_TMIPA) (20 mg, 0.12 mmol) and bipyridine (10 mg, 0.06 mmol) was suspended in 15 mL mixed solvents of N,N'-dimethylformamide, ethanol and H_2_O (*v*/*v* = 1:1:1), and heated in a Teflon-lined steel bomb at 373 K for 4 days. After cooling to room temperature, colorless crystals were collected, washed with ethanol several times, and dried in the air (yield: 47%, based on H_2_TMIPA).

### Refinement

H atoms were generated geometrically and were allowed to ride on their parent atoms in the riding model approximations with C—H = 0.93 (aromatic) and 0.96 Å (methyl), U_iso_(H) = 1.2U_eq_(C) for aromatic H atoms and 1.5U_eq_(C) for methyl H atoms.

### Crystal data

**Table:**

[Cd(C_11_H_10_O_4_)(C_10_H_8_N_2_)]	*F*(000) = 1904
*M**_r_* = 474.77	*D*_x_ = 1.687 Mg m^−^^3^
Orthorhombic, *P**b**c**a*	Mo *K*α radiation, λ = 0.71073 Å
Hall symbol: -P 2ac 2ab	Cell parameters from 5089 reflections
*a* = 13.1985 (8) Å	θ = 2.2–27.8°
*b* = 15.5714 (9) Å	µ = 1.20 mm^−^^1^
*c* = 18.1926 (11) Å	*T* = 298 K
*V* = 3738.9 (4) Å^3^	Block, colorless
*Z* = 8	0.15 × 0.10 × 0.10 mm

### Data collection

**Table:**

Bruker SMART APEXII CCD diffractometer	4299 independent reflections
Radiation source: fine-focus sealed tube	2454 reflections with *I* > 2σ(*I*)
graphite	*R*_int_ = 0.054
ω and φ scans	θ_max_ = 27.6°, θ_min_ = 2.2°
Absorption correction: multi-scan (*SADABS*; Bruker, 2001)	*h* = −17→11
*T*_min_ = 0.841, *T*_max_ = 0.890	*k* = −17→20
14349 measured reflections	*l* = −15→23

### Refinement

**Table:**

Refinement on *F*^2^	Primary atom site location: structure-invariant direct methods
Least-squares matrix: full	Secondary atom site location: difference Fourier map
*R*[*F*^2^ > 2σ(*F*^2^)] = 0.045	Hydrogen site location: inferred from neighbouring sites
*wR*(*F*^2^) = 0.098	H-atom parameters constrained
*S* = 0.99	*w* = 1/[σ^2^(*F*_o_^2^) + (0.0375*P*)^2^] where *P* = (*F*_o_^2^ + 2*F*_c_^2^)/3
4299 reflections	(Δ/σ)_max_ = 0.001
253 parameters	Δρ_max_ = 0.51 e Å^−^^3^
0 restraints	Δρ_min_ = −0.63 e Å^−^^3^

### Special details

**Table:**

Geometry. All esds (except the esd in the dihedral angle between two l.s. planes) are estimated using the full covariance matrix. The cell esds are taken into account individually in the estimation of esds in distances, angles and torsion angles; correlations between esds in cell parameters are only used when they are defined by crystal symmetry. An approximate (isotropic) treatment of cell esds is used for estimating esds involving l.s. planes.
Refinement. Refinement of F^2^ against ALL reflections. The weighted R-factor wR and goodness of fit S are based on F^2^, conventional R-factors R are based on F, with F set to zero for negative F^2^. The threshold expression of F^2^ > 2sigma(F^2^) is used only for calculating R-factors(gt) etc. and is not relevant to the choice of reflections for refinement. R-factors based on F^2^ are statistically about twice as large as those based on F, and R- factors based on ALL data will be even larger.

### Fractional atomic coordinates and isotropic or equivalent isotropic displacement parameters (Å^2^)

**Table:**

	*x*	*y*	*z*	*U*_iso_*/*U*_eq_	
Cd1	0.50573 (2)	0.584163 (18)	0.575068 (16)	0.03177 (11)	
C1	0.3060 (4)	0.7000 (3)	0.6257 (3)	0.0496 (14)	
H1A	0.3218	0.7291	0.5826	0.060*	
C2	0.2259 (4)	0.7293 (3)	0.6667 (3)	0.0569 (15)	
H2A	0.1873	0.7758	0.6512	0.068*	
C3	0.2046 (4)	0.6877 (3)	0.7314 (3)	0.0634 (16)	
H3A	0.1518	0.7064	0.7613	0.076*	
C4	0.2620 (4)	0.6187 (3)	0.7511 (3)	0.0586 (15)	
H4A	0.2489	0.5903	0.7951	0.070*	
C5	0.3391 (3)	0.5909 (3)	0.7063 (2)	0.0385 (11)	
C6	0.4024 (4)	0.5140 (3)	0.7238 (2)	0.0396 (11)	
C7	0.3857 (4)	0.4630 (3)	0.7852 (3)	0.0471 (13)	
H7A	0.3346	0.4768	0.8183	0.057*	
C8	0.4453 (4)	0.3916 (3)	0.7965 (3)	0.0541 (15)	
H8A	0.4352	0.3573	0.8377	0.065*	
C9	0.5189 (4)	0.3718 (3)	0.7472 (3)	0.0622 (16)	
H9A	0.5590	0.3232	0.7533	0.075*	
C10	0.5323 (5)	0.4249 (4)	0.6888 (3)	0.082 (2)	
H10A	0.5835	0.4116	0.6555	0.098*	
C11	0.6847 (3)	0.4899 (3)	0.5399 (2)	0.0311 (10)	
C12	0.7761 (3)	0.4466 (3)	0.5081 (2)	0.0335 (11)	
C13	0.8065 (3)	0.3639 (3)	0.5314 (2)	0.0344 (11)	
C14	0.8932 (3)	0.3269 (3)	0.5012 (3)	0.0387 (12)	
C15	0.9490 (4)	0.3712 (3)	0.4471 (3)	0.0581 (16)	
C16	0.9164 (4)	0.4521 (3)	0.4247 (3)	0.0699 (18)	
H16A	0.9529	0.4813	0.3888	0.084*	
C17	0.8299 (4)	0.4907 (3)	0.4549 (3)	0.0543 (15)	
C18	0.7974 (5)	0.5776 (3)	0.4262 (3)	0.078 (2)	
H18A	0.7381	0.5964	0.4522	0.117*	
H18B	0.7823	0.5733	0.3747	0.117*	
H18C	0.8511	0.6183	0.4334	0.117*	
C19	0.7417 (4)	0.3160 (3)	0.5853 (3)	0.0456 (13)	
H19A	0.7717	0.2611	0.5955	0.068*	
H19B	0.6753	0.3078	0.5648	0.068*	
H19C	0.7365	0.3483	0.6301	0.068*	
C20	0.9284 (3)	0.2395 (3)	0.5264 (3)	0.0417 (12)	
C21	1.0421 (5)	0.3344 (4)	0.4112 (4)	0.097 (3)	
H21A	1.0683	0.3748	0.3761	0.146*	
H21B	1.0249	0.2819	0.3866	0.146*	
H21C	1.0926	0.3232	0.4479	0.146*	
N1	0.3624 (3)	0.6324 (2)	0.64418 (19)	0.0367 (9)	
N2	0.4764 (3)	0.4950 (3)	0.6760 (2)	0.0532 (12)	
O1	0.6951 (3)	0.5405 (2)	0.59115 (17)	0.0536 (9)	
O2	0.5988 (2)	0.47532 (18)	0.51372 (16)	0.0412 (8)	
O3	0.9433 (3)	0.2269 (2)	0.5937 (2)	0.0580 (10)	
O4	0.9409 (3)	0.18136 (19)	0.48063 (19)	0.0618 (11)	

### Atomic displacement parameters (Å^2^)

**Table:**

	*U*^11^	*U*^22^	*U*^33^	*U*^12^	*U*^13^	*U*^23^
Cd1	0.0381 (2)	0.02748 (17)	0.02972 (17)	−0.00141 (17)	0.00347 (18)	−0.00099 (13)
C1	0.059 (4)	0.047 (3)	0.043 (3)	0.003 (3)	0.007 (3)	0.009 (2)
C2	0.056 (4)	0.048 (3)	0.067 (4)	0.008 (3)	0.011 (3)	0.005 (3)
C3	0.061 (4)	0.063 (4)	0.066 (4)	0.016 (3)	0.021 (3)	0.002 (3)
C4	0.063 (4)	0.059 (3)	0.054 (3)	0.017 (3)	0.019 (3)	0.017 (3)
C5	0.040 (3)	0.041 (3)	0.035 (3)	−0.001 (3)	0.006 (2)	−0.001 (2)
C6	0.044 (3)	0.038 (3)	0.038 (3)	−0.001 (2)	0.002 (2)	0.002 (2)
C7	0.051 (3)	0.047 (3)	0.044 (3)	0.004 (3)	0.015 (3)	0.011 (2)
C8	0.060 (4)	0.047 (3)	0.054 (4)	−0.011 (3)	0.002 (3)	0.021 (3)
C9	0.068 (4)	0.048 (3)	0.070 (4)	0.019 (3)	0.009 (3)	0.022 (3)
C10	0.098 (5)	0.065 (4)	0.082 (5)	0.040 (4)	0.044 (4)	0.033 (3)
C11	0.029 (3)	0.026 (2)	0.038 (3)	0.004 (2)	0.002 (2)	0.007 (2)
C12	0.033 (3)	0.027 (2)	0.040 (3)	0.000 (2)	−0.003 (2)	0.005 (2)
C13	0.025 (3)	0.031 (2)	0.047 (3)	−0.004 (2)	−0.006 (2)	0.001 (2)
C14	0.026 (3)	0.030 (2)	0.061 (3)	0.003 (2)	0.003 (2)	0.001 (2)
C15	0.041 (3)	0.044 (3)	0.089 (4)	0.010 (3)	0.021 (3)	0.010 (3)
C16	0.055 (4)	0.048 (3)	0.106 (5)	0.014 (3)	0.040 (4)	0.034 (3)
C17	0.045 (3)	0.039 (3)	0.079 (4)	0.014 (3)	0.015 (3)	0.009 (3)
C18	0.084 (5)	0.049 (3)	0.102 (5)	0.020 (3)	0.036 (4)	0.029 (3)
C19	0.035 (3)	0.038 (3)	0.064 (3)	0.000 (2)	0.000 (3)	0.008 (2)
C20	0.034 (3)	0.027 (3)	0.064 (4)	0.002 (2)	−0.002 (3)	0.004 (3)
C21	0.069 (4)	0.071 (4)	0.152 (7)	0.034 (4)	0.063 (5)	0.032 (4)
N1	0.044 (3)	0.030 (2)	0.036 (2)	−0.002 (2)	0.0083 (19)	0.0022 (17)
N2	0.065 (3)	0.046 (2)	0.048 (3)	0.019 (2)	0.022 (2)	0.016 (2)
O1	0.049 (2)	0.061 (2)	0.051 (2)	0.0074 (19)	−0.0078 (18)	−0.0182 (18)
O2	0.0239 (18)	0.0478 (19)	0.052 (2)	0.0033 (16)	−0.0045 (16)	−0.0085 (15)
O3	0.072 (3)	0.038 (2)	0.064 (3)	0.0192 (19)	−0.023 (2)	−0.0055 (17)
O4	0.092 (3)	0.0328 (19)	0.061 (2)	0.017 (2)	0.010 (2)	−0.0006 (17)

### Geometric parameters (Å, °)

**Table:**

Cd1—N1	2.393 (4)	C11—O2	1.250 (5)
Cd1—N2	2.334 (4)	C11—C12	1.499 (6)
Cd1—O1	2.607 (3)	C12—C17	1.382 (6)
Cd1—O2^i^	2.317 (3)	C12—C13	1.414 (5)
Cd1—O2	2.372 (3)	C13—C14	1.394 (6)
Cd1—O3^ii^	2.347 (3)	C13—C19	1.500 (6)
Cd1—O4^ii^	2.396 (3)	C14—C15	1.409 (6)
C1—N1	1.333 (5)	C14—C20	1.510 (6)
C1—C2	1.371 (6)	C15—C16	1.392 (7)
C1—H1A	0.9300	C15—C21	1.505 (7)
C2—C3	1.372 (6)	C16—C17	1.402 (7)
C2—H2A	0.9300	C16—H16A	0.9300
C3—C4	1.363 (7)	C17—C18	1.514 (6)
C3—H3A	0.9300	C18—H18A	0.9600
C4—C5	1.374 (6)	C18—H18B	0.9600
C4—H4A	0.9300	C18—H18C	0.9600
C5—N1	1.338 (5)	C19—H19A	0.9600
C5—C6	1.494 (6)	C19—H19B	0.9600
C6—N2	1.341 (5)	C19—H19C	0.9600
C6—C7	1.388 (6)	C20—O4	1.241 (5)
C7—C8	1.377 (6)	C20—O3	1.255 (5)
C7—H7A	0.9300	C20—Cd1^iii^	2.718 (4)
C8—C9	1.357 (7)	C21—H21A	0.9600
C8—H8A	0.9300	C21—H21B	0.9600
C9—C10	1.359 (7)	C21—H21C	0.9600
C9—H9A	0.9300	O2—Cd1^i^	2.317 (3)
C10—N2	1.339 (6)	O3—Cd1^iii^	2.347 (3)
C10—H10A	0.9300	O4—Cd1^iii^	2.396 (3)
C11—O1	1.228 (5)		
			
O2^i^—Cd1—N2	102.23 (14)	O1—C11—O2	120.5 (4)
O2^i^—Cd1—O3^ii^	130.54 (12)	O1—C11—C12	119.5 (4)
N2—Cd1—O3^ii^	119.81 (14)	O2—C11—C12	120.0 (4)
O2^i^—Cd1—O2	72.22 (12)	C17—C12—C13	121.1 (4)
N2—Cd1—O2	91.79 (12)	C17—C12—C11	117.4 (4)
O3^ii^—Cd1—O2	126.60 (12)	C13—C12—C11	121.5 (4)
O2^i^—Cd1—N1	91.21 (11)	C14—C13—C12	119.4 (4)
N2—Cd1—N1	69.02 (13)	C14—C13—C19	121.4 (4)
O3^ii^—Cd1—N1	81.58 (12)	C12—C13—C19	119.1 (4)
O2—Cd1—N1	151.76 (11)	C13—C14—C15	120.2 (4)
O2^i^—Cd1—O4^ii^	85.85 (11)	C13—C14—C20	120.3 (4)
N2—Cd1—O4^ii^	171.39 (14)	C15—C14—C20	119.5 (4)
O3^ii^—Cd1—O4^ii^	54.62 (11)	C16—C15—C14	119.0 (5)
O2—Cd1—O4^ii^	87.81 (12)	C16—C15—C21	118.0 (5)
N1—Cd1—O4^ii^	114.27 (12)	C14—C15—C21	123.0 (5)
O2^i^—Cd1—O1	123.00 (10)	C15—C16—C17	121.6 (5)
N2—Cd1—O1	85.17 (13)	C15—C16—H16A	119.2
O3^ii^—Cd1—O1	87.47 (12)	C17—C16—H16A	119.2
O2—Cd1—O1	50.95 (10)	C12—C17—C16	118.7 (4)
N1—Cd1—O1	141.36 (11)	C12—C17—C18	122.7 (4)
O4^ii^—Cd1—O1	87.91 (12)	C16—C17—C18	118.6 (5)
O2^i^—Cd1—C20^ii^	108.60 (14)	C17—C18—H18A	109.5
N2—Cd1—C20^ii^	146.97 (16)	C17—C18—H18B	109.5
O3^ii^—Cd1—C20^ii^	27.47 (12)	H18A—C18—H18B	109.5
O2—Cd1—C20^ii^	108.48 (13)	C17—C18—H18C	109.5
N1—Cd1—C20^ii^	98.32 (13)	H18A—C18—H18C	109.5
O4^ii^—Cd1—C20^ii^	27.16 (12)	H18B—C18—H18C	109.5
O1—Cd1—C20^ii^	87.82 (12)	C13—C19—H19A	109.5
N1—C1—C2	123.8 (4)	C13—C19—H19B	109.5
N1—C1—H1A	118.1	H19A—C19—H19B	109.5
C2—C1—H1A	118.1	C13—C19—H19C	109.5
C1—C2—C3	117.9 (5)	H19A—C19—H19C	109.5
C1—C2—H2A	121.1	H19B—C19—H19C	109.5
C3—C2—H2A	121.1	O4—C20—O3	121.3 (4)
C4—C3—C2	118.9 (5)	O4—C20—C14	119.6 (5)
C4—C3—H3A	120.5	O3—C20—C14	119.0 (4)
C2—C3—H3A	120.5	O4—C20—Cd1^iii^	61.8 (2)
C3—C4—C5	120.3 (5)	O3—C20—Cd1^iii^	59.6 (2)
C3—C4—H4A	119.9	C14—C20—Cd1^iii^	178.4 (4)
C5—C4—H4A	119.9	C15—C21—H21A	109.5
N1—C5—C4	121.2 (4)	C15—C21—H21B	109.5
N1—C5—C6	116.1 (4)	H21A—C21—H21B	109.5
C4—C5—C6	122.7 (4)	C15—C21—H21C	109.5
N2—C6—C7	120.8 (4)	H21A—C21—H21C	109.5
N2—C6—C5	116.4 (4)	H21B—C21—H21C	109.5
C7—C6—C5	122.8 (4)	C1—N1—C5	117.8 (4)
C8—C7—C6	119.4 (5)	C1—N1—Cd1	123.9 (3)
C8—C7—H7A	120.3	C5—N1—Cd1	118.3 (3)
C6—C7—H7A	120.3	C10—N2—C6	117.9 (4)
C9—C8—C7	119.6 (5)	C10—N2—Cd1	122.0 (3)
C9—C8—H8A	120.2	C6—N2—Cd1	120.0 (3)
C7—C8—H8A	120.2	C11—O1—Cd1	88.5 (3)
C10—C9—C8	118.1 (5)	C11—O2—Cd1^i^	151.4 (3)
C10—C9—H9A	120.9	C11—O2—Cd1	99.2 (3)
C8—C9—H9A	120.9	Cd1^i^—O2—Cd1	107.78 (12)
N2—C10—C9	124.1 (5)	C20—O3—Cd1^iii^	93.0 (3)
N2—C10—H10A	118.0	C20—O4—Cd1^iii^	91.1 (3)
C9—C10—H10A	118.0		

Symmetry codes: (i) −*x*+1, −*y*+1, −*z*+1; (ii) −*x*+3/2, *y*+1/2, *z*; (iii) −*x*+3/2, *y*−1/2, *z*.

### Hydrogen-bond geometry (Å, °)

**Table:**

*D*—H···*A*	*D*—H	H···*A*	*D*···*A*	*D*—H···*A*
C4—H4A···O1^iv^	0.93	2.32	3.240 (6)	169
C8—H8A···O3^iv^	0.93	2.39	3.251 (6)	155

Symmetry codes: (iv) *x*−1/2, *y*, −*z*+3/2.
